# Tristetraprolin suppresses the EMT through the down-regulation of Twist1 and Snail1 in cancer cells

**DOI:** 10.18632/oncotarget.7094

**Published:** 2016-01-31

**Authors:** Nal Ae Yoon, Hyun Gun Jo, Unn Hwa Lee, Ji Hye Park, Ji Eun Yoon, Jinhyun Ryu, Sang Soo Kang, Young Joo Min, Seong-A Ju, Eun Hui Seo, In Young Huh, Byung Ju Lee, Jeong Woo Park, Wha Ja Cho

**Affiliations:** ^1^ Department of Biological Sciences, University of Ulsan, Ulsan 680–749, Korea; ^2^ Department of Anatomy and Convergence Medical Science, Institute of Health Sciences, School of Medicine, Gyeongsang National University, Jinju, Gyeongnam 52727, Korea; ^3^ Department of Internal Medicine, Ulsan University Hospital, University of Ulsan College of Medicine, Ulsan 682-060, Korea; ^4^ Biomedical Research Center, Ulsan University Hospital, University of Ulsan College of Medicine, Ulsan 682-060, Korea; ^5^ Department of Anesthesiology and Pain Medicine, Ulsan University Hospital, University of Ulsan College of Medicine, Ulsan 682–060, Korea

**Keywords:** tristetraprolin, EMT, Twist1, Snail1, cell migration

## Abstract

Inhibition of epithelial-mesenchymal transition (EMT)-inducing transcription factors Twist and Snail prevents tumor metastasis but enhances metastatic growth. Here, we report an unexpected role of a tumor suppressor tristetraprolin (TTP) in inhibiting *Twist* and *Snail* without enhancing cellular proliferation. TTP bound to the AU-rich element (ARE) within the mRNA 3′UTRs of *Twist1* and *Snail1*, enhanced the decay of their mRNAs and inhibited the EMT of cancer cells. The ectopic expression of *Twist1* or *Snail1* without their 3′UTRs blocked the inhibitory effects of TTP on the EMT. We also observed that TTP overexpression suppressed the growth of cancer cells. Our data propose a new model whereby TTP down-regulates *Twist1* and *Snail1* and inhibits both the EMT and the proliferation of cancer cells.

## INTRODUCTION

The EMT is a normal developmental program that promotes epithelial cell dissociation and migration to different sites during embryogenesis [1, 2]. During cancer progression, tumor cells often gain the ability to activate the EMT to migrate away from the primary tumor and invade the surrounding stromal tissues [3]. During the EMT, cells down-regulate epithelial markers such as E-cadherin and catenins [4] and express mesenchymal markers including N-cadherin, vimentin, and fibronectin [5]. The EMT process is induced by a group of transcription factors including the zinc finger factors Snail and ZEB and the basic helix-loop-helix factor Twist [6]. As crucial EMT inducers, Twist1 and Snail1 are up-regulated in many types of cancer and are associated with the increased invasive behavior of cancer cells [7]. Twist and Snail increase the pathogenesis of several malignant neoplasms, predominantly by enhancing invasiveness and metastatic behavior. The exogenous overexpression of Twist1 or Snail1 increases the invasive and metastatic abilities of cancer cells by promoting the down-regulation of E-cadherin and the induction of an EMT [8–10]. However, EMT-inducing factors can also inhibit the proliferation of cancer cells [11–13]. Thus, once they reach distant sites, cancer cells need to down-regulate the EMT-inducing factors to allow for metastatic growth [13]. At the transcriptional level, the expression of EMT-inducing factors is up-regulated by developmental signal transduction pathways, such as transforming growth factor β (TGF-β), Notch, and fibroblast growth factor [1]. However, even though several microRNAs have been reported to inhibit *Twist1* and *Snail1* mRNAs [7, 14], little is known about the post-transcriptional regulation of these genes.

AU-rich elements (AREs) post-transcriptionally regulate the expression of a variety of short-lived mRNAs such as cytokines and proto-oncogenes [15]. The stability of ARE-containing mRNAs is regulated by ARE-binding proteins [16]. One of the best-characterized ARE-binding proteins is tristetraprolin (TTP), which promotes the degradation of ARE-containing transcripts [17, 18]. TTP expression is significantly decreased in various cancers [19]. The decreased TTP expression correlates with the increased expression of proto-oncogenes and may contribute to cancer processes and the re-expression of TTP induces growth inhibitory effects [20–22]. TTP expression is induced by p53 in cancer cells [23]. However, nearly all types of cancers have abnormalities in the p53 pathway [24], which may explain the widespread decrease in TTP in human cancers.

We show here for the first time that the expression of TTP led to a decrease in EMT markers and the migration of cancer cells. TTP did not decrease the mRNA stability of EMT markers but enhanced the mRNA degradation of the EMT inducers *Twist1* and *Snail1*. The exogenous expression of either *Twist1* or *Snail1* without the 3′UTR recovered the expression of EMT markers and cell migration. These studies thus indicate a novel signaling pathway by which TTP inhibits EMT and cell migration through the down-regulation of both *Twist1* and *Snail1* at the post-transcriptional level. It has been reported that the inhibition of EMT-inducing factors promotes growth in cancer cells [13]. However, TTP did not promote cancer cell growth but instead suppressed cellular proliferation through the down-regulation of genes involved in cell proliferation such as *c-fos*, *CDC34*, and *VEGF*. These findings suggest that TTP serves as a negative regulator of both metastasis and proliferation in cancer cells.

## RESULTS

### TTP suppresses the mesenchymal phenotype in cancer cells

**Figure 1:**
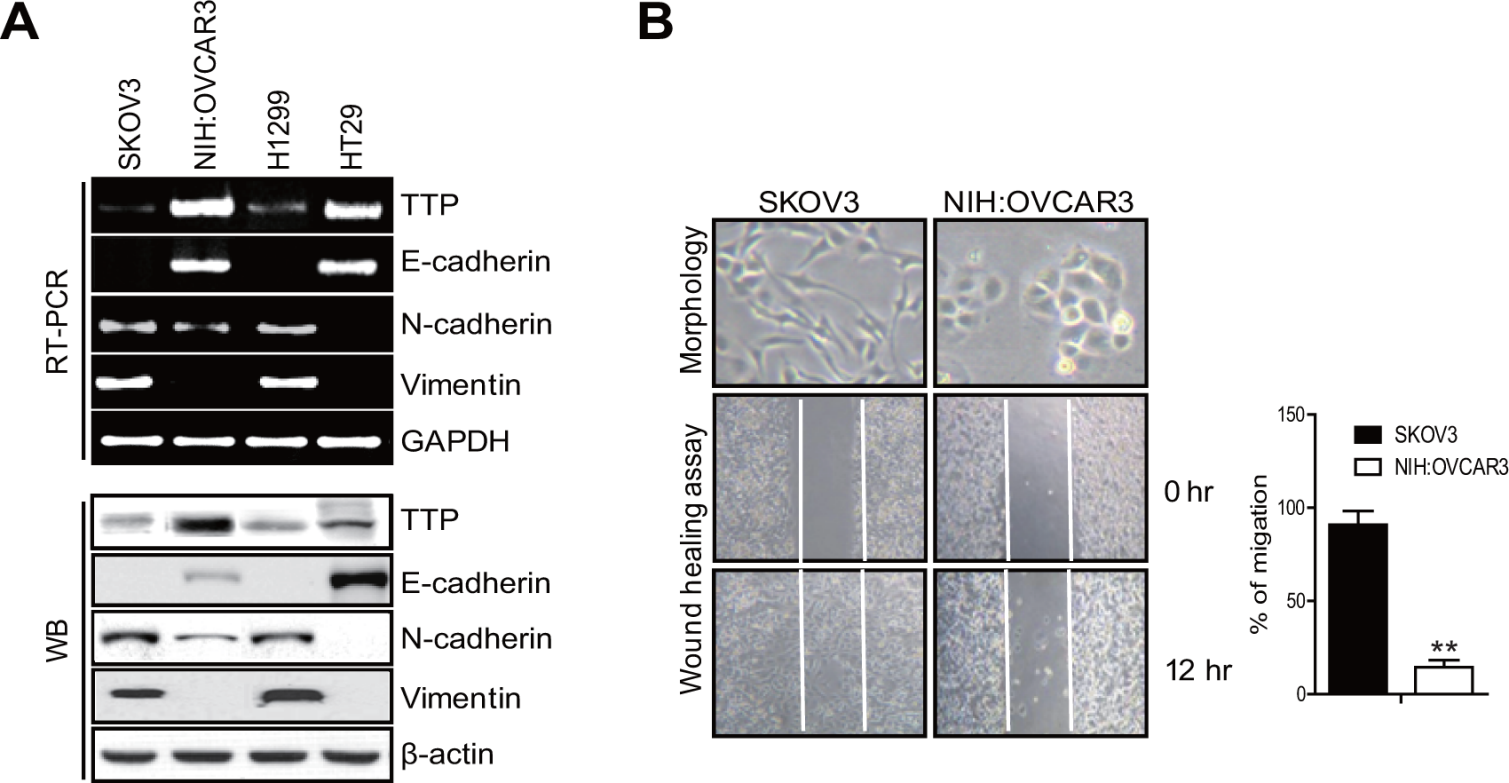
Cancer cells with a low TTP level show a mesenchymal phenotype (**A**) The levels of TTP and EMT markers in the cancer cells. The levels of *TTP*, *E-cadherin*, *N-cadherin*, and *vimentin* were determined by semi-qRT-PCR (top) and Western blot (bottom) in SKOV3, NIH:OVCAR3 (ovarian adenocarcinoma), HT29 (colorectal adenocarcinoma), and H1299 (non-small lung carcinoma) cancer cell lines. SKOV3 cells with low TTP expression and NIH:OVCAR3 cells with high TTP expression were selected for further study. (**B**) Cell morphology and wound-healing assay. Cell morphology (top) and the wounded areas (bottom) of SKOV3 and NIH:OVCAR3 cells were examined under x100 and x20 magnification, respectively. Data are representative of three experiments. Data are presented as the mean *±* SD (*n* = 3) (**p < 0.01).

Previously, we reported that the overexpression of TTP suppresses cellular proliferation by enhancing the mRNA degradation of oncogenes [20, 25]. In addition, we found that the cancer cells that overexpressed TTP underwent a dramatic shape change, becoming smaller and establishing more cell-cell contacts. This prompted us to investigate the possible role of TTP in the regulation of EMT. We first analyzed ovarian (SKOV3 and NIH:OVCAR3), colon (HT29), and lung (H1299) cancer cell lines for the expression of *TTP*, the epithelial marker *E-cadherin*, and the mesenchymal markers *N-cadherin* and *vimentin* by RT-PCR and Western blot. The NIH:OVCAR3 and HT29 cells expressed high levels of *TTP* and *E-cadherin* but low levels of *N-cadherin* and *vimentin* (Figure 1A). In SKOV3 and H1299 cells, the levels of *TTP* and *E-cadherin* were low but those of *N-cadherin* and *vimentin* were high. These data suggest that *TTP* expression in these cancer cell lines is positively correlated with the epithelial marker *E-cadherin* but negatively correlated with the mesenchymal markers *N-cadherin* and *vimentin* (Figure 1A).

**Figure 2:**
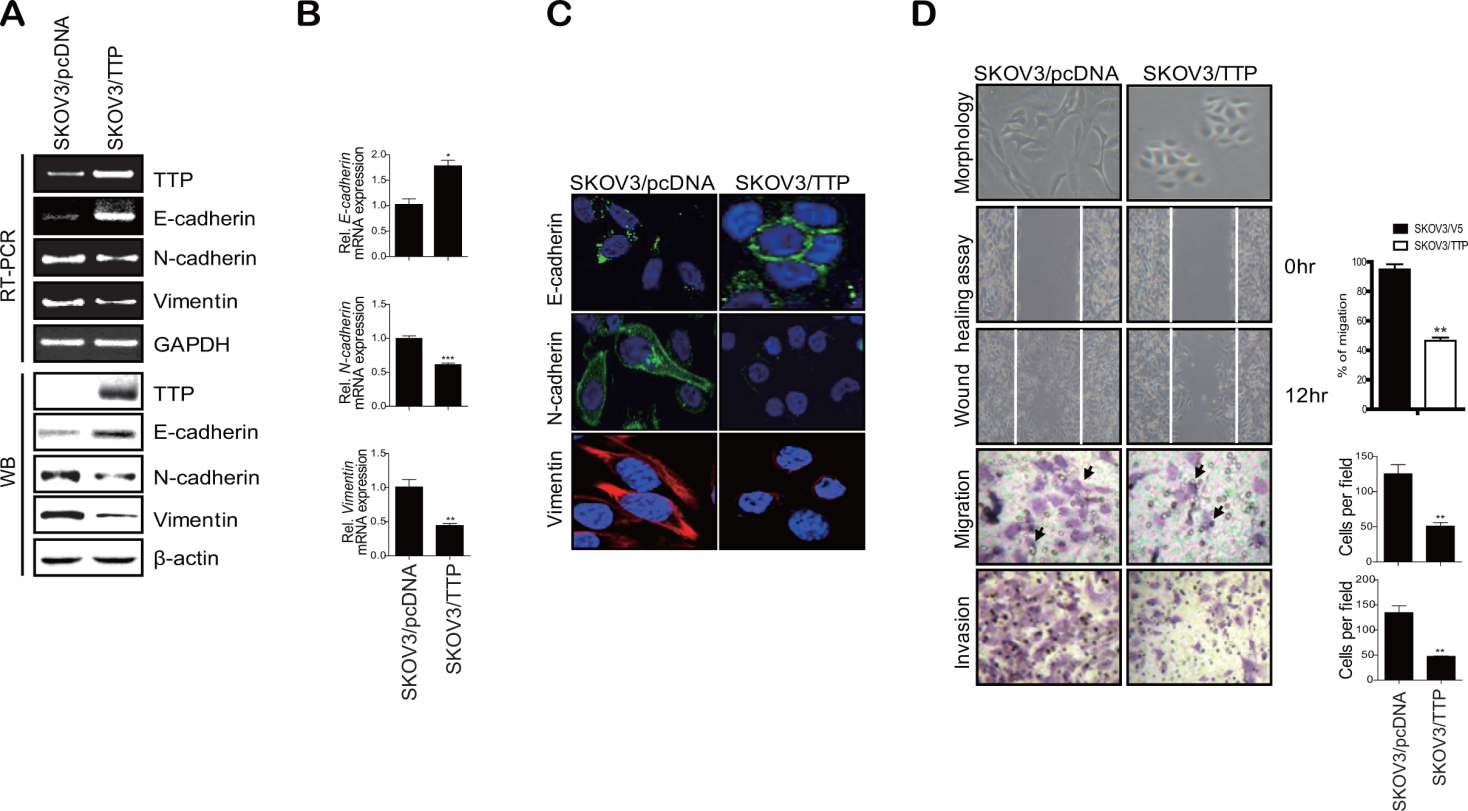
TTP overexpression induces a shift from a mesenchymal to an epithelial phenotype in human cancer cells 2 × 10^6^ SKOV3 cells were transiently transfected with 1 μg pcDNA6/V5-TTP (SKOV3/TTP) or empty vector pcDNA6/V5 (SKOV3/pcDNA) for 24 h. The levels of *TTP*, *E-cadherin*, *N-cadherin*, and *vimentin* were determined by semi-qRT-PCR ((**A**) top), Western blot (A, bottom), qRT-PCR (**B**), and immunofluorescent staining (**C**). Data are presented as the mean *±* SD (*n* = 3) (*p < 0.05; **p < 0.01; ***p < 0.001). (**D**) Cell morphology, wound-healing assay, migration, and invasion. Cell morphology (top) and the wounded areas (2nd) of SKOV3/pcDNA and SKOV3/TTP cells were examined under x100 and x20 magnification, respectively. Data are representative of three experiments. Data are presented as the mean *±* SD (*n* = 3) (**p < 0.01). The migration (3rd) and invasion (bottom) of SKOV3/pcDNA and SKOV3/TTP cells were determined by the trans-well chamber assay. Arrows indicate cells. Data are representative of three experiments. Data are presented as the mean *±* SD (*n* = 3) (**p < 0.01).

**Figure 3:**
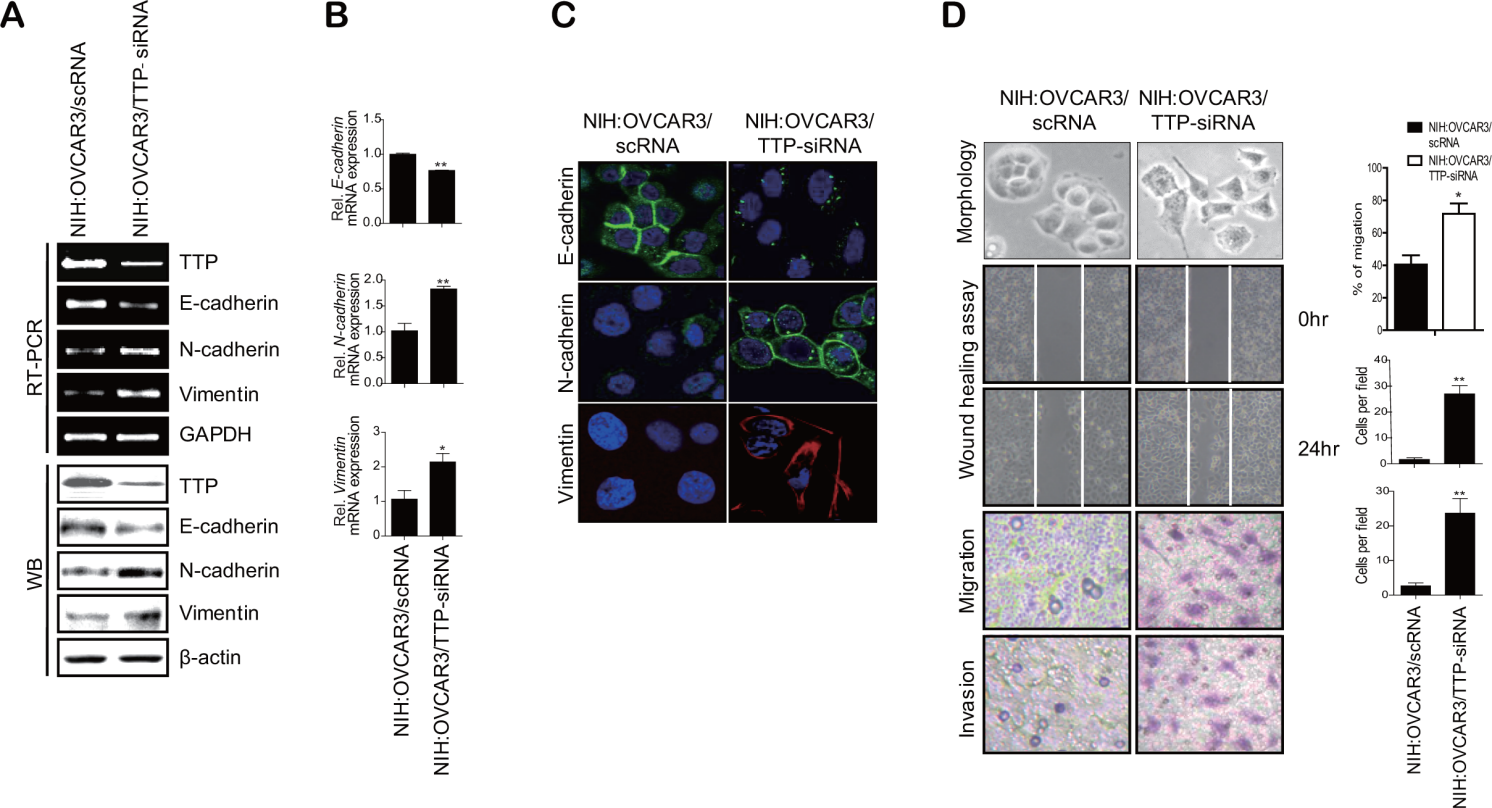
Down-regulation of TTP by siRNA induces a shift from the epithelial to mesenchymal phenotype in human cancer cells 2 × 10^6^ NIH:OVCAR3 cells were transfected with 60 pmol scRNA or TTP-specific siRNA (TTP-siRNA) for 24 h. The levels of TTP, *E-cadherin*, *N-cadherin*, and *vimentin* were determined by semi-qRT-PCR ((**A**) top), Western blot (A, bottom), qRT-PCR (**B**), and immunofluorescent staining (**C**). Data are presented as the mean *±* SD (*n* = 3) (*p < 0.05; **p < 0.01). (**D**) Wound-healing assay, migration, and invasion. The wounded areas (top) of NIH:OVCAR3/scRNA and NIH:OVCAR3/TTP-siRNA cells were examined under × 20 magnification. Data are representative of three experiments. Data are presented as the mean *±* SD (*n* = 3) (*p < 0.05). The migration (2nd) and invasion (3rd) of NIH:OVCAR3/scRNA and NIH:OVCAR3/TTP-siRNA cells were determined by the trans-well chamber assay. Data are representative of three experiments. Data are presented as the mean *±* SD (*n* = 3) (*p < 0.05).

In order to determine whether TTP inhibits the EMT, we selected two ovarian cancer cell lines: SKOV3 with low TTP expression and NIH:OVCAR3 with high TTP expression. These two cell lines showed differences in cell morphology and motility. While SKOV3 showed an extensively flattened and elongated leading-trailing mesenchymal morphology, NIH:OVCAR3 showed a small epithelial morphology (Figure 1B). In addition, SKOV3 cells migrated faster than NIH:OVCAR3 cells in the wound healing assay (Figure 1B). We tested the effect of TTP overexpression on the EMT. SKOV3 cells were transfected with pcDNA6/V5-TTP (SKOV3/TTP) or the control pcDNA/V5 vector (SKOV3/pcDNA), and we analyzed the levels of the EMT markers by RT-PCR, Western blot, and immunofluorescent staining. TTP overexpression in SKOV3 cells increased *E-cadherin* but decreased *N-cadherin* and *vimentin* (Figure 2A–2C). We also determined the effects of TTP overexpression on cell morphology and migration using a wound healing assay, and trans-well migration and invasion assay. The ectopic expression of TTP induced a transition from elongated mesenchymal morphology to small epithelial morphology (Figure 2D, top). In both the wound healing assay and trans-well migration assay, TTP overexpression suppressed the migration and invasion of SKOV3 cells (Figure 2D, middle and bottom). Next, we determined the effects of TTP inhibition on the EMT. NIH:OVCAR3 cells were transfected with siRNA against TTP in order to inhibit the expression of TTP, and we analyzed for the expression of EMT markers by RT-PCR, Western blot, and immunofluorescent staining. The inhibition of *TTP* by siRNA decreased the levels of *E-cadherin* but increased the levels *N-cadherin* and *vimentin* (Figure 3A–3C). The inhibition of *TTP* enhanced cell migration in the wound healing assay and the trans-well migration and invasion assay (Figure 3D). Collectively, our data suggest that TTP suppresses EMT in human ovarian cancer cells.

### TTP does not affect the mRNA stabilities of EMT markers

TTP has been reported to control gene expression by enhancing the degradation of target mRNAs [17, 18]. To determine whether TTP affects the stability of *E-cadherin*, *N-cadherin*, or *vimentin* mRNA, the half-lives of these mRNAs were calculated from the mRNA levels measured by qRT-PCR in the SKOV3 cells transfected with pcDNA6/V5-TTP or a control pcDNA6/V5 vector. In both the control SKOV3/pcDNA and the SKOV3/TTP cells, these mRNAs were stable for 6 h after actinomycin D treatment (Supplementary Figure S1), indicating that TTP does not directly affect the expression of the EMT markers *E-cadherin*, *N-cadherin*, or *vimentin* in cancer cells.

### TTP destabilizes the mRNAs of the EMT-inducing transcription factors *Twist1* and *Snail1*

**Figure 4:**
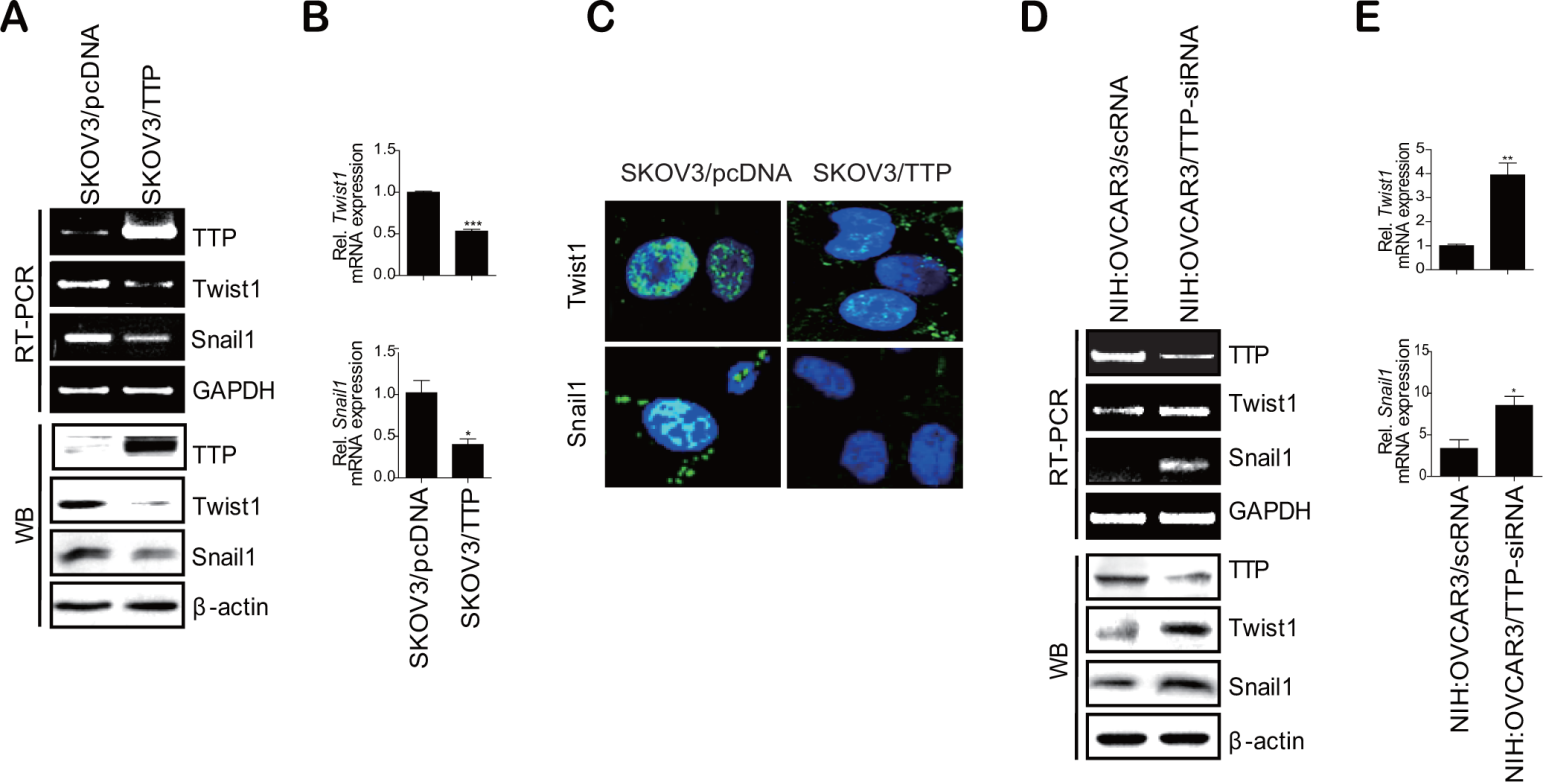
TTP inhibits the expression of *Twist1* and *Snail1* in human cancer cells (**A–C**) Overexpression of *TTP* inhibits the levels of *Twist1* and *Snail1* in SKOV3 cells. 2 × 10^6^ SKOV3 cells were transfected with 1 μg pcDNA6/V5-TTP (SKOV3/TTP) or pcDNA6/V5 (SKOV3/pcDNA) for 24 h. (A) The levels of *TTP*, *Twist1*, and *Snail1* were determined by semi-qRT-PCR (A, top), Western blot (A, bottom), qRT-PCR (B), and immunofluorescent staining (C). Data are representative of three experiments. Data are presented as the mean *±* SD (*n* = 3) (*p < 0.05; ***p < 0.001). (**D**–**E**) The downregulation of *TTP* by siRNA increases the levels of *Twist1* and *Snail1* in NIH:OVCAR3 cells. NIH:OVCAR3 cells were transfected with scRNA or TTP-specific siRNA (TTP-siRNA) for 24 h. D. The levels of *TTP*, *Twist1*, and *Snail1* were determined by semi-qRT-PCR (D, top), Western blot (D, bottom), and qRT-PCR (E). Data are representative of three experiments. Data are presented as the mean *±* SD (*n* = 3) (*p < 0.05; **p < 0.01).

**Figure 5:**
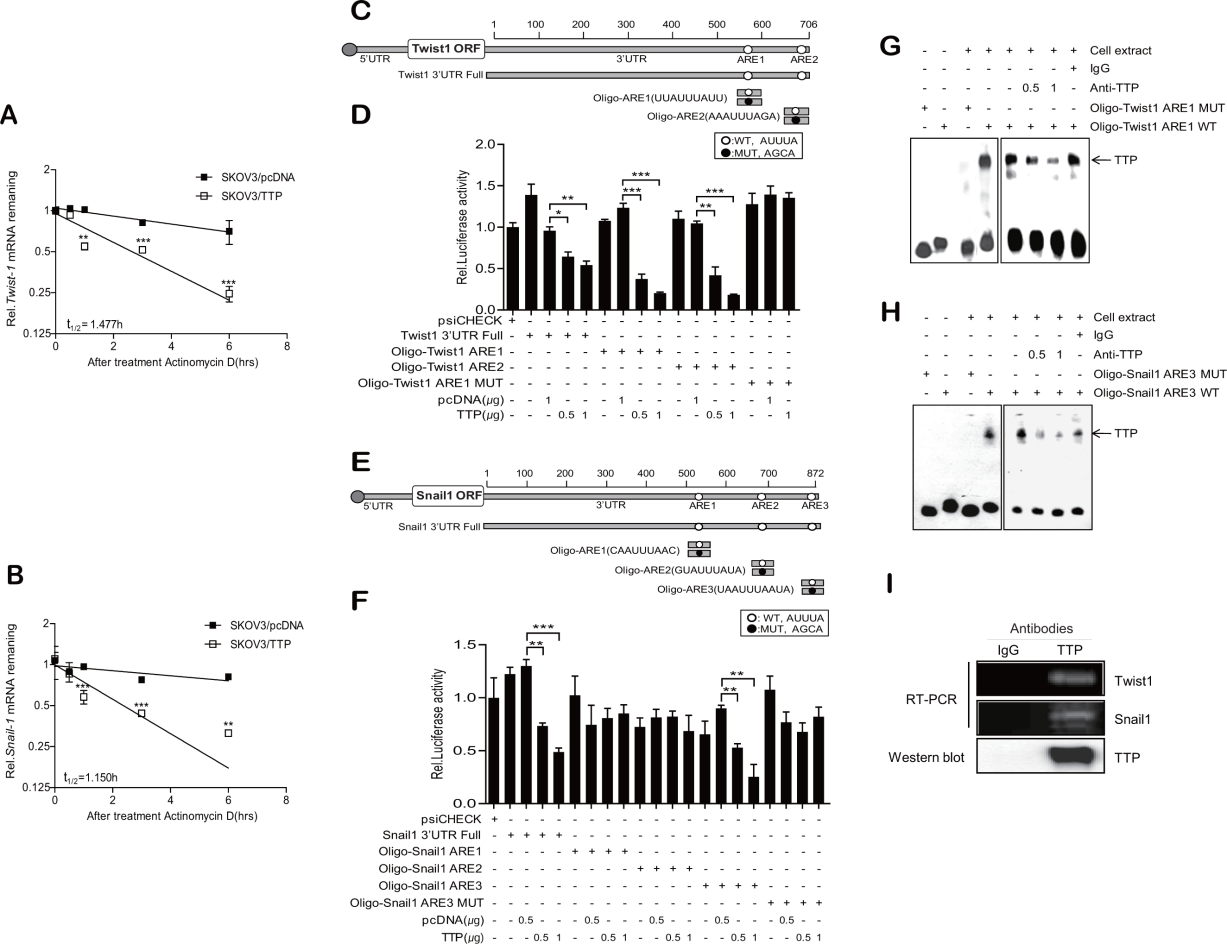
TTP enhances the decay of *Twist1* and *Snail1* mRNA through binding with an ARE within their mRNA 3′UTRs (**A**–**B**) TTP destabilizes the mRNAs of *Twist1* and *Snail1*. 2 × 10^6^ SKOV3 cells were transfected with 1 μg pcDNA6/V5-TTP or pcDNA6/V5 for 24 h. The expression of *Twist1* (A) and *Snail1* (B) mRNAs in SKOV3 cells was determined by qRT-PCR at the indicated times after the addition of 5 μg/ml actinomycin D. Data are presented as the mean *±* SD (*n* = 3) (**p < 0.01; ***p < 0.001). (**C**–**F**) The first or the second AUUUA pentamer (ARE1 and ARE2, respectively) within the *Twist1* 3′UTR and the third AUUUA pentamer (ARE3) within the *Snail1* 3′UTR are necessary for the inhibitory effect of TTP. C–E. Schematic representation of the luciferase reporter constructs used in this study. Fragments and oligonucleotides (Oligo) derived from the *Twist1* (C) or *Snail1* (E) mRNA 3′UTR were cloned downstream of the luciferase reporter gene in the psiCHECK2 luciferase expression vector. White circles, wild-type (WT) pentameric motif AUUUA; black circles, mutated (MUT) motif AGCA. D–F. SKOV3 cells were co-transfected with pcDNA6/V5-TTP and psiCHECK2 luciferase reporter constructs containing oligonucleotides derived from the *Twist1* or *Snail1* mRNA 3′UTR as described in (C and E) for 24 h. After normalizing for luciferase activity, the luciferase activity obtained from the SKOV3 cells transfected with the psiCHECK2 vector alone were set to 1.0. Data are presented as the mean *±* SD (*n* = 3) (**p < 0.01; ***p < 0.001). G–H. RNA EMSA was performed by mixing cytoplasmic extracts containing 5 μg of total protein from pcDNA6/V5-TTP-transfected SKOV3 cells with 20 fmol biotinylated wild-type Oligo-ARE (WT) or mutant Oligo-ARE (MUT) probe: Oligo-ARE1 for *Twist1*; Oligo-ARE3 for *Snail1*. Anti-TTP or control antibody was added to the reaction mixtures. The positions of the TTP-containing bands (TTP) are indicated. (**I**) Ribonucleoprotein immunoprecipitation assay. The ribonucleoprotein complexes containing TTP in NIH:OVCAR3 cells were immunoprecipitated with protein G-agarose and anti-TTP or a control antibody. The Twist1 or Snail1 mRNA in the immunoprecipitates was amplified by RT-PCR. The presence of TTP in the immunoprecipitates was detected by western blot with anti-TTP antibody.

The expression of EMT markers is induced by EMT-inducing transcription factors such as Twist1, Twist2, Snail1, Snail2, ZEB1, and ZEB2 [6]. Thus, we hypothesized that TTP controls the expression of EMT markers by inhibiting the expression of EMT-inducing transcription factors. To test this hypothesis, we analyzed the expression of these EMT-inducing transcription factors in SKOV3/pcDNA and SKOV3/TTP by RT-PCR and Western blot. TTP overexpression did not decrease the expression of *Twist2*, *Snail2*, *ZEB1*, or *ZEB2* (Supplementary Figure S2A and S2B). Even though the expression of *Snail2* decreased in SKOV3/TTP, the stability of *Snail2* mRNA was not decreased by TTP overexpression (Supplementary Figure S2C). However, the expression of *Twist1* and *Snail1* was reduced in SKOV3/TTP cells compared with that in SKOV3/pcDNA cells (Figure 4A–4C). H1299 cells with ectopic expression of TTP also showed decreased expression of both *Twist1* and *Snail1* (Supplementary Figure S3). In order to confirm whether the expression of Twist1 and Snail1 is controlled by TTP, we determined the effect of TTP down-regulation on the expression of these transcription factors. NIH:OVCAR3 and HT29 cells with high level of TTP were used for this study. The inhibition of TTP by siRNA (TTP-siRNA) increased the expression of both *Twist1* and *Snail1*(Figure 4D, 4E, and Supplementary Figure S3). To determine whether TTP controls the stability of *Twist1* and *Snail1* mRNAs, the half-lives of their mRNAs were calculated from the mRNA levels measured by qRT-PCR in SKOV3/TTP and SKOV3/pcDNA cells. In the control SKOV3/pcDNA cells, both *Twist1 and Snail1* mRNAs were stable for 6 h after actinomycin D treatment. However, in SKOV3/TTP cells, the half-lives of *Twist1* and *Snail1* mRNAs decreased to 1.47 h and 1.15 h, respectively (Figure 5A and 5B). Collectively, these results suggest that TTP down-regulates the expression of both *Twist1* and *Snail1* in cancer cells.

### TTP decreases the expression of luciferase mRNA containing the *Twist1* or *Snail1* 3′UTR

TTP protein decreases mRNA stability by binding AREs within the mRNA 3′UTR [17, 20]. There are three classes in AREs: class I ARE contains scattered AUUUA pentameric motifs with a nearby U-rich region; class II ARE, at least two overlapping copies of the uuAUUUAuu nonamer in a U-rich region; class III, U-rich regions [26]. Analysis of the human *Twist1 and Snail1* mRNA 3′UTRs revealed the presence of a class I ARE and a class II ARE in *Twist1* 3′UTR and three class I AREs in *Snail1* 3′UTR (Figure 5C and 5E). To determine whether the down-regulation of *Twist1* and *Snail1* expression by TTP is mediated through their mRNA 3′UTRs, we used a luciferase reporter gene linked to *Twist1* 3′UTR containing ARE1 (the first ARE of *Twist1* 3′UTR, class II ARE) and ARE2 (the second ARE of *Twist1* 3′UTR, class I ARE), *Snail1* 3′UTR containing three class I AREs (ARE1, the first ARE; ARE2 the second ARE; ARE3, the third ARE of *Snail1* 3′UTR), and oligonucleotides containing the respective AREs of *Twist1* and *Snail1* 3′UTRs: Oligo-Twist1 ARE1, Oligo-Twist1 ARE2, Oligo-Snail1 ARE1, Oligo-Snail1 ARE2, and Oligo-Snail1 ARE3. We also prepared luciferase reporter genes containing mutant oligonucleotides with each AUUUA motif of the *Twist1* and *Snail1* 3′UTRs (ARE-MUT, containing AUUUA sequences substituted with AGCA). When SKOV3 cells were transfected to overexpress TTP, luciferase activity from *Twist1* 3′UTR, Oligo-Twist1 ARE1, Oligo-Twist1 ARE2, Snail1 3′UTR, and Oligo-Snail1 ARE3 was inhibited; however, Oligo-Snail1 ARE1 and Oligo-Snail1 ARE2 did not respond to TTP (Figure 5D and 5F). In addition, Oligo-Twist1 ARE1-MUT and Oligo-Snail1 ARE3-MUT did not respond to TTP (Figure 5D and 5F). The results suggest that both the first and the second AUUUA motifs within the *Twist1* 3′UTR and the third AUUUA motif within the *Snail1* 3′UTR are responsible for the destabilization of *Twist1 and Snail1* mRNAs, respectively, by TTP.

### TTP binds to the AUUUA motif within the *Twist1* and *Snail1* mRNA 3′UTRs

To determine the binding of TTP with the ARE of the *Twist1* and *Snail1* 3′UTRs, RNA EMSA was conducted using a biotinylated RNA probe containing the wild-type or mutant ARE1 of *Twist1* or *Snail1*. The RNA probes used for RNA EMSA were the same as those used for the luciferase assay. Cytoplasmic extracts were prepared from SKOV3/TTP cells and incubated with biotinylated RNA probes containing the wild-type or mutant ARE1 of the *Twist1* or *Snail1* 3′UTR. When RNA EMSA was conducted using the wild-type ARE1 probe of *Twist1* or the wild-type ARE3 of *Snail1*, a dominant RNA-protein complex was observed; however, the mutant ARE1 of *Twist1* and the mutant ARE3 of *Snail1* failed to form the complex. Complex formation was neutralized in the presence of an anti-TTP antibody (Figure 5G and 5H). These results demonstrate that TTP binds specifically with the AREs of *Twist1* and *Snail1*.

To confirm the binding between TTP with the AREs of *Twist1* or *Snail1* 3′UTR, the ribonucleoprotein complexes containing TTP in NIH:OVCAR3 cells were immunoprecipitated with anti-TTP or a control antibody. Total RNA was extracted from the immunoprecipitates and the presence of Twist1 or Snail1 mRNA was analyzed by RT-PCR using PCR primers specific to the *Twist1* 3′UTR or *Snail1* 3′UTR. The amplified PCR product was observed in immunoprecipitates obtained using anti-TTP antibody (Figure 5I). PCR product was not also detected in immunoprecipitates obtained using control antibody. Taken together, these data strongly suggest that the repression of *Twist1* and *Snail1* occurs through the binding of TTP with the ARE of the *Twist1* and *Snail1* 3′UTRs.

### Overexpression of *Twist1* or *Snail1* attenuates the inhibitory effects of TTP on the mesenchymal phenotype

**Figure 6:**
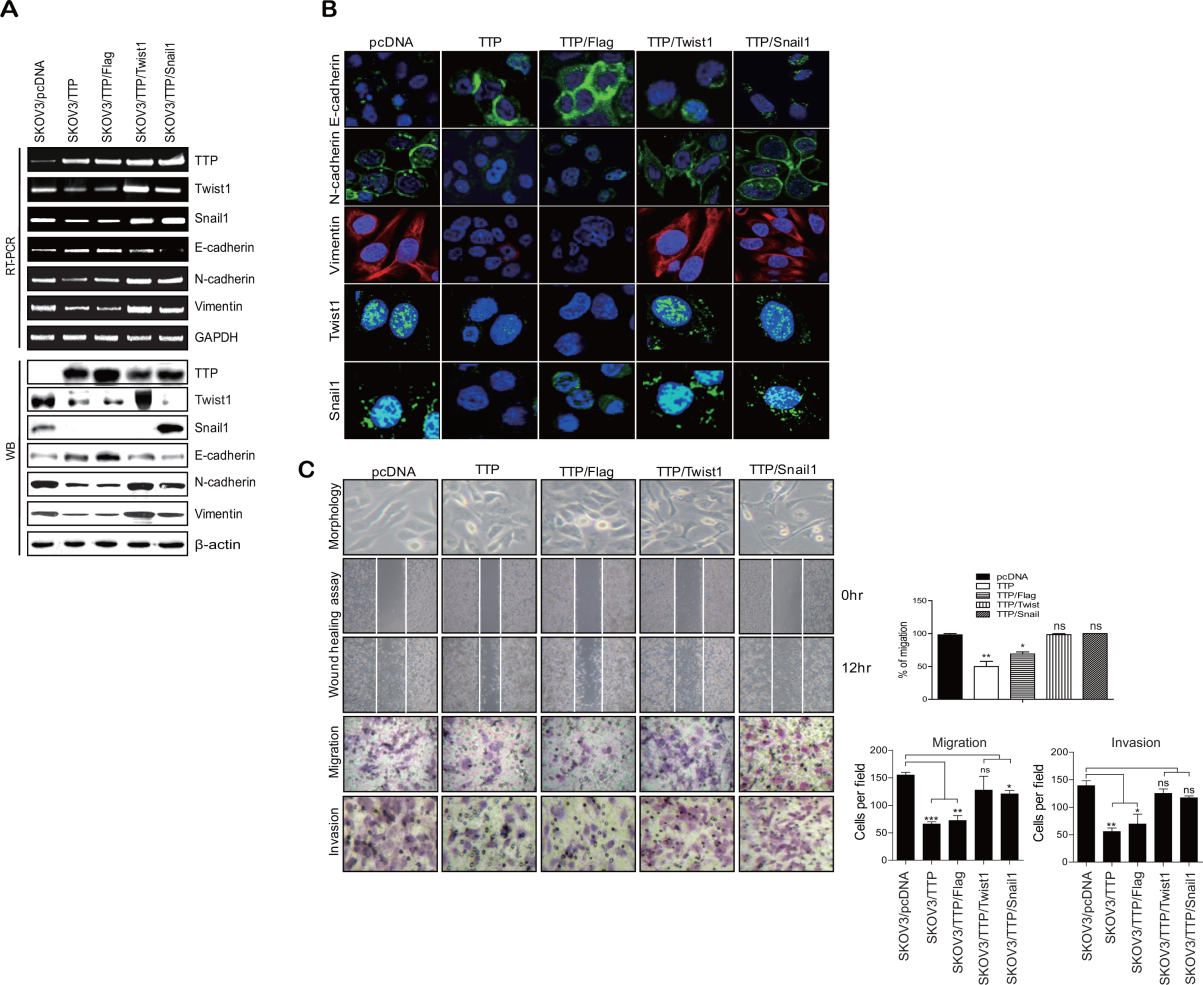
Overexpression of Twist1 or Snail1 cDNA without the 3′UTR attenuates the inhibitory effect of TTP on the mesenchymal phenotype 2 × 10^6^ SKOV3 cells were transfected with a combination of 0.5 μg pcDNA6/V5-TTP and 0.5 μg pCMV/Flag-Twist1 or pCMV/Flag-Snail1 for 24 h (**A** and **B**). The levels of *TTP*, *Twist1*, *Snail1*, *E-cadherin*, *N-cadherin*, and *vimentin* were measured by semi-qRT-PCR (A, top), Western blot (A, bottom), and immunofluorescent staining (B). (**C**) Cell morphology, wound-healing assay, migration, and invasion. Cell morphology (top) and the wounded areas (2nd) of SKOV3 transfected with a combination of pcDNA6/V5-TTP and pCMV/Flag-Twist1 or pCMV/Flag-Snail1 were examined under x100 and x20 magnification, respectively. Data are representative of three experiments. Data are presented as the mean *±* SD (*n* = 3) (*p < 0.05; **p < 0.01). ns, not significant. The migration (3rd) and invasion (bottom) of the SKOV3 cells were determined by the trans-well chamber assay. Data are representative of three experiments. Data are presented as the mean *±* SD (*n* = 3) (*p < 0.05; **p < 0.01; ***p < 0.001). ns, not significant.

Based on our results, it was speculated that TTP controls the expression levels of EMT markers through the down-regulation of *Twist1* and *Snail1*. To confirm this hypothesis, we co-transfected SKOV3 cells with pcDNA6/V5-TTP and pCMV/Flag-Twist1 or pCMV/Flag-Snail1, which do not contain a 3′UTR. At 24-h post-transfection, cells were analyzed for the expression of *E-cadherin*, *N-cadherin*, and *vimentin* and for cell migration. The overexpression of *Twist1* or *Snail1* abrogated the effect of TTP on the expression of *E-cadherin*, *N-cadherin*, and *vimentin* (Figure 6A and 6B). In addition, the overexpression of *Twist1* or *Snail1* restored the migration and the invasion of SKOV3 cells (Figure 6C). These results indicate that *TTP* suppresses the EMT through the down-regulation of the EMT inducers *Twist1* or *Snail1* in cancer cells.

### TTP level is high in the epithelium but low in the mesenchyme of human tissues

Previously we reported that TTP level is high in normal tissues but significantly reduced in tumor stage I and remains at very low levels in advanced stages of human colonic adenocarcinoma [20] and ovarian adenocarcinoma [25]. Based on our results, it was speculated that TTP level is high in the normal epithelium but low in the normal mesenchyme. To confirm this hypothesis, we determined the expression levels of TTP in normal human ovarian tissues and colonic mucosa by immunohistochemistry. Consistent with our hypothesis, we found that, while normal ovarian tissues and normal colonic mucosa demonstrated strong TTP staining of the surface epithelium and the mesenchyme of normal ovarian tissues and colonic mucosa was negative for TTP (Supplementary Table S2 and Supplementary Figure S4).

### TGF-β does not decrease the expression of TTP in cancer cells

Transforming growth factor β (TGF-β) has been reported to induce EMT in cancer cells [27] and induce TTP expression [28, 29]. Thus, we tested whether TGF-β induces EMT by modulating TTP expression in cancer cells. We found that TGF-β inhibited the expression of *E-cadherin* and enhanced the expression of *Twist1*, *Snail1*, and *N-cadherin* in A549 cells (Supplementary Figure S5). To determine whether these factors induce these changes by inhibiting TTP, A549 cells were incubated in the presence of TGF-β for 24 h and analyzed for the expression of TTP. However, TGF-β treatment did not decrease the expression of TTP (Supplementary Figure S5A and S5B). In addition, we found that TGF-β treatment did not enhance the mRNA stability of *Twist1*, *E-cadherin*, *N-cadherin*, and *vimentin* (Supplementary Figure S5C). These results suggest that TTP does not mediate the TGF-β-induced EMT in cancer cells.

### Doxorubicin induces TTP expression and inhibits EMT

Ectopic expression of TTP was found to inhibit EMT in cancer cells (Figure 2). We next asked whether a TTP-inducer inhibits EMT in cancer cells. Previously, we have reported that the DNA-damaging agent doxorubicin (DOX) induces the expression of TTP in a p53-dependent manner [23]. Thus, we hypothesized that DOX induces TTP expression, which in turn decreases the expression of EMT markers. To test this hypothesis, we used p53 wild-type PA1 ovarian cancer cells instead of p53 mutant NIH:OVCAR3 and SKOV3 cells. PA1 cells were treated with DOX for 24 h and analyzed for the expression of TTP and EMT markers. High concentration of DOX is toxic to cells and thus we used low concentration of DOX (0.3 μg/ml) to prevent DOX-induced cell death. As shown in Supplementary Figure S6A, DOX treatment increased the expression of *TTP* and *E-cadherin* but decreased the levels of *Twist1*, *N-cadherin*, and *vimentin*. DOX treatment did not decrease the mRNA stability of E-cadherin, N-cadherin, and vimentin but enhanced the degradation of Twist1 mRNA (Supplementary Figure S6B). In addition, DOX treatment inhibited cellular migration in the wound healing assay (Supplementary Figure S6C). These results suggest that the TTP inducer DOX can inhibit the EMT in cancer cells.

### TTP inhibits the growth of ovarian cancer cells

**Figure 7:**
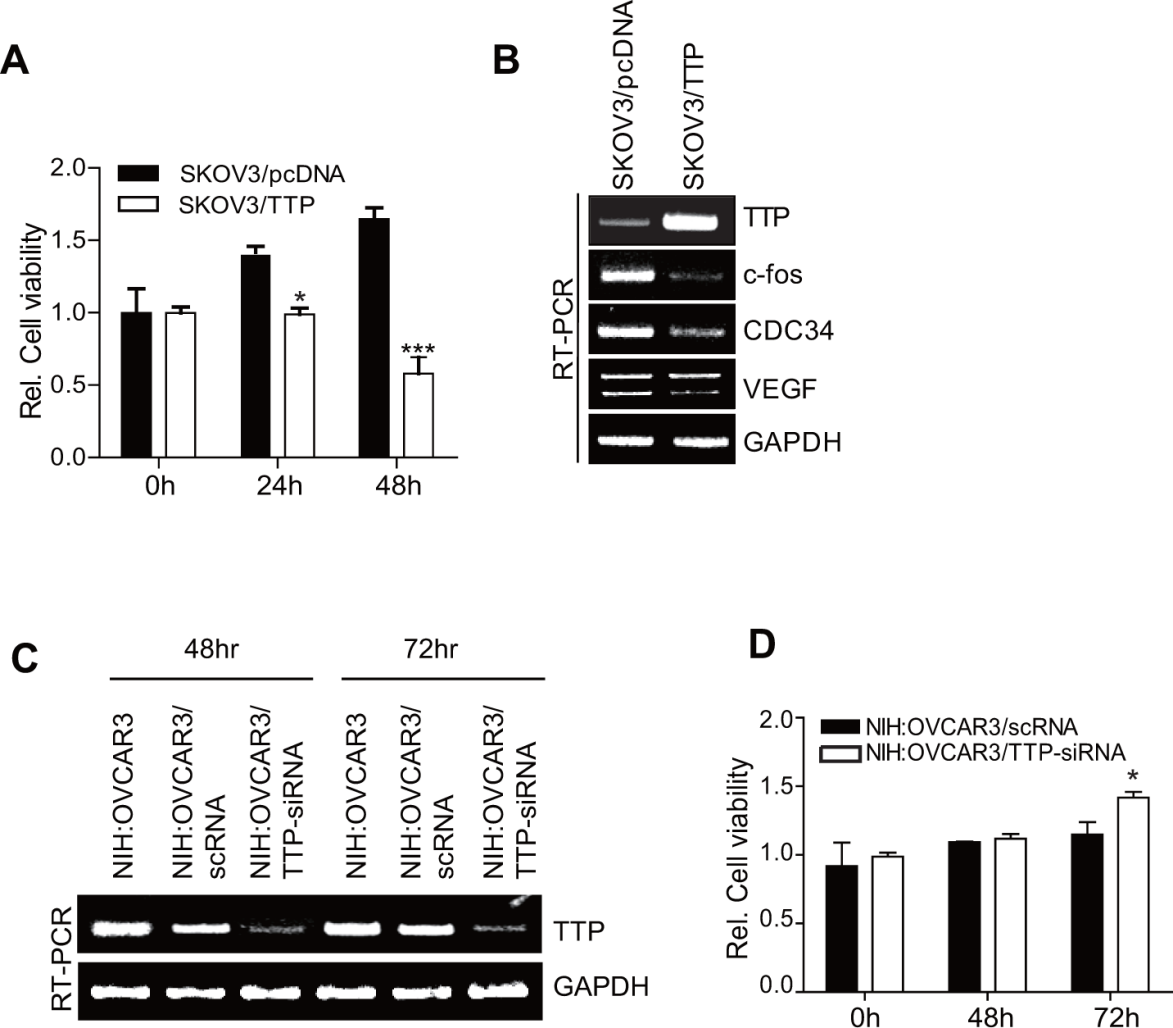
TTP suppresses the growth of cancer cells (**A–B**) Overexpression of TTP suppresses the growth of cancer cells. 2 × 10^6^ SKOV3 cells were transiently transfected with 1 μg pcDNA6/V5-TTP (SKOV3/TTP) or empty vector pcDNA6/V5 (SKOV3/pcDNA) for 24 h. (A) Cell viability was assessed at indicated times after transfection by measuring absorbance at 490 nm using a MTS cell proliferation assay. Data are presented as the mean *±* SD (*n* = 3) (*p < 0.05; ***p < 0.001). (B) The levels of *TTP*, *c-fos*, *CDC34*, and *VEGF* were determined at 48 h after transfection by semi-qRT-PCR. (**C**–**D**) The downregulation of *TTP* by siRNA increases the growth of cancer cells. NIH:OVCAR3 cells were transfected with scRNA or TTP-specific siRNA (TTP-siRNA) for 24 h. **C.** The level of *TTP* was determined at indicated time after transfection by semi-qRT-PCR. (D) Cell viability was assessed at indicated time after transfection by measuring absorbance at 490 nm using a MTS cell proliferation assay. Data are presented as the mean *±* SD (*n* = 3) (*p < 0.05).

The inhibition of EMT-inducing factors has been reported to promote cancer cell growth [13]. We tested whether TTP overexpression enhances the growth of cancer cells. Consistent with our previous reports [20, 25], the ectopic expression of TTP inhibited the growth of SKOV3 cells (Figure 7A). RT-PCR analysis revealed a decreased expression of *c-fos*, *CDC34*, and *VEGF* in TTP-overexpressing SKOV3 cells (Figure 7B). On the contrary, the inhibition of TTP by siRNA (Figure 7C) enhanced the growth of NIH:OVCAR3 cells (Figure 7D). These results indicate that TTP inhibits the expression of oncogenes as well as the EMT-inducing factors *Twist1* and *Snail1* and suppresses both the growth and metastasis of cancer cells.

## DISCUSSION

Previously TTP has been reported to be involved in tumor metastasis [30, 31]. However, the underlying mechanism remains unknown. The present study identified TTP, a RNA-binding protein that inhibits the EMT in human cancer cells. TTP overexpression in human cancer cells also increased the levels of the epithelial marker *E-cadherin* but decreased the mesenchymal markers *N-cadherin* and *vimentin*. Conversely, the inhibition of TTP by siRNA decreased the *E-cadherin* level but increased *N-cadherin* and *vimentin* levels. We also observed that TTP overexpression suppresses cell migration, while TTP inhibition enhances it. In normal human tissues, the levels of TTP expression were high in the epithelium but extremely low in the mesenchyme. Together, this evidence leads us to propose that TTP is a negative regulator of the EMT.

TTP has been reported to inhibit gene expression at the post-transcriptional level through enhancing the degradation of ARE-containing mRNAs [17, 18]. If EMT markers such as *E-cadherin*, *N-cadherin*, and *vimentin* are target genes of TTP in cancer cells, TTP might decrease the stability of the mRNA of these genes. However, we found that TTP did not decrease the stability of these mRNAs, bringing into question the identities of the targets of TTP in regulating the EMT phenotype. The expression of EMT marker genes is controlled by several EMT inducers such as Twist1, Twist2, Snail1, Snail2, ZEB1, and ZEB2 [6]. In this study, we demonstrated that TTP down-regulates *Twist1* and *Snail1* expression in cancer cells, suggesting the possibility that TTP may inhibit the expression of EMT marker genes through enhancing the degradation of *Twist1* and *Snail1* mRNAs. Here, we provided evidence supporting this hypothesis: overexpression of TTP decreased mRNA stability and the expression levels of *Twist1* and *Snail1*; inhibition of TTP by siRNA increased expression levels of *Twist1* and *Snail1*; ectopic expression of *Twist1* or *Snail1* cDNA without 3′UTR could revert the EMT phenotype in TTP-overexpressing cancer cells; TTP bound to the ARE motif within the 3′UTRs of *Twist1* and *Snail1*; and TTP inhibited the expression of a luciferase reporter gene containing the 3′UTR of *Twist1* or *Snail1*. Our results indicate that TTP down-regulates *Twist1* and *Snail1* expression at the post-transcriptional level by enhancing the degradation of their mRNAs in order to inhibit EMT marker gene expression and the EMT phenotype.

As crucial EMT inducers, Twist1 and Snail1 are up-regulated in many types of cancer and are associated with increased invasive behavior in cancer cells [7]. Twist1 and Snail1 are up-regulated by a wide range of signaling pathways [1]. Among them, TGF-β is one of the most prominent EMT-inducing cytokines that activate an array of EMT inducers [32]. In this study, we demonstrated that TTP inhibits the expression of Twist1 and Snail1 in human cancer cells. TTP expression has been reported to be inhibited in a variety of human cancer cells [19], which may cause a high level of Twist1 and Snail1 to be expressed in cancer cells. However, it is not likely that an EMT inducer such as TGF-β is responsible for the low TTP levels in cancer cells since TGF-β has been reported to induce TTP expression [28, 29] and enhance TTP activity [33]. In addition, we did not observe any changes in TTP expression in the cancer cells that had undergone an EMT upon stimulation with TGF-β. Previously, we have identified p53 to be a key transcription factor in inducing TTP expression in human cancer cells [23]. The DNA-damaging agent doxorubicin has been reported to induce the expression of TTP in p53 wild-type cancer cells [23]. In this study, we observed that doxorubicin enhanced the *TTP* level and decreased *Twist1* and *Snail1* levels in p53 wild-type cancer cells. This suggests that the re-expression of *TTP* inhibits the EMT through the down-regulation of *Twist1* and *Snail1*. Until now, it has been reported that *TTP* expression is controlled by signaling pathways involving phorbol ester, insulin, serum, and other mitogenic stimuli [34–38]. Therefore, future studies are needed to identify TTP inducers that contribute to the down-regulation of EMT-inducing factors and metastasis.

Cancer cells undergoing EMT are often growth-arrested since EMT-inducing factors can inhibit the proliferation of cancer cells [11–13]. Thus, once reaching distant sites, cancer cells need to down-regulate EMT-inducing factors to allow for metastatic growth. The underlying mechanisms, however, are largely unknown. If TTP expression is enhanced during cancer progression, it is possible that TTP is responsible for the deactivation of EMT-inducing factors that support metastatic growth. However, the enhanced expression of TTP has not been observed during cancer progression [20, 25], suggesting that TTP may not be responsible for the deactivation of EMT-inducing factors to promote metastatic growth. Here, we found that, in normal tissues, TTP level is high in the epithelium but low in the mesenchyme. In this normal epithelium, TTP may suppress the EMT through down-regulation of EMT-inducing factors such as *Twist1* and *Snail1*. However, the deactivation of Twist1 and Snail1 may not lead to enhanced cellular proliferation since TTP can inhibit growth through the down-regulation of cancer-associated genes [39, 40]. Consistent with these previous studies, we found that high levels of TTP induced by ectopic expression or doxorubicin treatment inhibited the proliferation of cancer cells. Collectively, our results suggest that TTP in the epithelium suppresses the EMT through the down-regulation of EMT-inducing factors such as *Twist1* and *Snail1* without enhancing cellular proliferation.

In conclusion, we determined that TTP suppresses the EMT of human cancer cells through the destabilization of *Twist1* and *Snail1* mRNAs. These findings, coupled with recent evidence demonstrating the benefits of EMT in cancer metastasis [1], suggest that pharmacologic activation of TTP and/or the induction of TTP expression may limit EMT and cancer metastasis. Therapeutic agents that inhibit EMT have been proposed as a treatment option against tumor metastasis [41]. However, such an approach could promote metastatic growth when patients present with circulating cancer cells. TTP can suppress both EMT and cellular proliferation through the down-regulation of EMT-inducing factors and cancer-related genes, respectively. Thus, the TTP pathway could represent a new therapeutic target to prevent both metastasis and cellular proliferation.

## MATERIALS AND METHODS

### Cell culture

Human ovarian (SKOV3, NIH:OVCAR3, PA1), colon (HT29), and lung (H1299, A549) cancer cell lines were purchased from the Korean Cell Line Bank (KCLB-Seoul, Korea). Cells were cultured in RPMI1 1640 media, supplemented with 10% fetal bovine serum (WELGENE, Korea), and were maintained at 37°C in a humidified at atmosphere of 5% CO_2_. To determine the effect of TGF-β on TTP expression and the EMT, cells were incubated with 10 ng/ml human TGF-β (Sigma, SRP3170) for 24 h. For the induction of TTP, cells were treated with 0.3 μg/ml Doxorubicin (DOX) (Sigma, D1515) for 24 h.

### Cell migration and invasion assays

A wound-healing assay was conducted using a culture-insert (ibidi, 80209) according to the manufacturer′s instruction. After 24 h, the culture insert was removed, leaving gaps (approx. 500 μm) in the sheets of cells. The area devoid of cells was analyzed after 12 or 24 h using a Carl Zeiss microscope (OLYMPUS, CK30).

For the trans-well migration assay, cells were re-suspended in serum-free medium and seeded into the trans-well inserts of a 24-well plate (BD Biosciences, #3422) for 24 h. The culture medium containing 10% fetal bovine serum was placed in the lower chamber as a chemoattractant. After incubation for 24 h, non-migrated cells were scraped off the upper surface of the membrane with a cotton swab. Migrated cells remaining on the bottom surface were counted after staining with crystal violet (0.5% in 20% methanol). The invasion assay was performed under the same conditions using growth factor-reduced matrigel-coated insert wells (BD Biosciences, #356234). Values for cell migration or invasion were expressed as the average number of cells per microscopic field over four fields per one filter for triplicate experiments, as described previously.

### Plasmids, siRNAs, transfections, and dual-luciferase assay

The pcDNA6/V5-TTP construct was described previously [25]. The plasmid construct pCMV/Flag-Twist1 was kindly provided by Dr. Kou-Juey Wu (Institute of Biochemistry and Mol Biology, National Yang-Ming University, Taiwan) and pCMV/Flag-Snail1 was purchased from Addgene. Cells were transfected with plasmid constructs using TurboFect^™^ *in vitro* transfection reagent (Thermo Scientific, R0531).

Small interfering RNAs (siRNAs) against human TTP (TTP-siRNA, sc-36760) and control siRNA (scRNA, sc-44230) were purchased from Santa Cruz Biotechnology (Santa Cruz). 2 × 10^6^ cells were transfected 24 h after plating with 60 pmol siRNA using Lipofectamine^™^ RNAiMAX (Invitrogen) and were harvested after 48 h. The expression levels of TTP or EMT marker mRNAs and proteins were analyzed by RT-PCR or Western blot.

Full length of Twist1 3′UTR and Snail1 3′UTR containing all AUUUA motifs of each 3′UTRs were PCR amplified from cDNA of SKOV3 cells using the following primer sets: Twist1–3′UTR-U, Twist1–3′UTR-D, Snail1–3′UTR-U, and Snail1–3′UTR-D (Supplementary Table S1). Two oligonucleotides containing the first (Oligo-Twist1 ARE1) and the second AUUUA pentamers (Oligo-Twist1 ARE2) within the *Twist1* 3′UTR and three oligonucleotides containing the first (Oligo-Snail1 ARE1), second (Oligo-Snail1 ARE2), and third AUUUA pentamers (Oligo-Snail1 ARE3) within the *Snail1* 3′UTR were synthesized at Integrated DNA Technologies (Coralville, IA, USA). Mutant oligonucleotides in which the AUUUA pentamer was substituted with AGCA (Oligo-ARE MUT) were also synthesized (Supplementary Table S1). The oligonuclotides were ligated into the XhoI/NotI site of the psiCHECK2 Renilla/firefly dual-luciferase expression vector (Promega, Madison, WI, USA).

For the luciferase assays, 2 × 10^6^ cells were co-transfected with 0.5 μg various psiCHECK2-Twist1 3′UTR and psiCHECK2-Snail1 3′UTR constructs and 0.5 μg pcDNA6/V5-TTP using the TurboFect^™^ *in vitro* transfection reagent (Thermo Scientific, R0531). Transfected cells were lysed with lysis buffer and mixed with luciferase assay reagent (Promega, 017757), and the chemiluminescent signal was measured in a SpectraMax L Microplate (Molecular Devices, Sunnyvale, CA, USA). Firefly luciferase was normalized to Renilla luciferase in each sample. All luciferase assays reported here represent at least three independent experiments, each consisting of three wells per transfection.

### Cell viability/proliferation

For the MTS cell proliferation assay, cells were plated in triplicate at 1.0 *×* 10^4^ cells/well in 96-well culture plates in culture media. At 24 or 48 h after plating, CellTiter 96^®^ AQueous One Solution cell proliferation assay (Promega, 3580) was added to each well according to the manufacturer's instructions, and absorbance at 490 nm was determined for each well using a Victor 1420 Multilabel Counter (EG&G Wallac, Turku, Finland).

### Electrophoretic mobility shift assay (EMSA)

The biotinylated RNA probes for the wild-type (Twist1-ARE1 WT, 5′-ACUUAAAAUACAAAAAACAACAUUCUAUUUAUUUAUUGAGGACCCAUGGUA AAAUGCAAA-3′; Snail1-ARE3 WT, 5′-GUUAUAUGUACAGUUUAUUGAUAUUCAAUAAAGCAGUUAAU UUAUAUAUUAAAAA-3′) and mutant (Twist1-ARE1 MUT, 5′-ACUUAAAAUACAAAAAACAACAUUCUA GCAGCAUUGAGGACCCAUGGUAAAAUGCAAA-3′; Snail1-ARE3 MUT, 5′-GUUAUAUGUACAGUUUAUUGAUAUUCAAUAAAGCAGUUAAGCAUAUAUUA AAAA-3′) cells were synthesized by ST Pharm Co., Ltd. (Seoul, Korea). A mutant RNA probe in which two AUUUA pentamers were each substituted with AGCA was used as a negative control. Cytoplasmic extracts were prepared from SKOV3 cells or pcDNA6/V5-TTP-transfected SKOV3 cells using NE-PER nuclear and cytoplasmic extraction reagent (Pierce, 78835). RNA EMSA was performed using the LightShift Chemiluminescent EMSA kit (Pierce, 20148x) according to the manufacturer's instructions. Briefly, 20 fmol of biotinylated RNA was combined with 5 μg of cytoplasmic protein from cell extracts in binding buffer. For the supershift EMSA, rabbit anti-human TTP polyclonal antibody (Sigma, T5327) or control antibody (Sigma, T3581) was added to the reaction mixture. After addition of the antibodies, the reaction mixtures were incubated overnight on ice and resolved on 5% non-denaturing polyacrylamide gels in 0.5X Tris borate/EDTA buffer. Gels were transferred to a nylon membrane (Hybond^™^-N^+^) in 0.5x Tris borate/EDTA at 70V for 40 min. Transferred RNAs were cross-linked to the membrane and detected using horseradish peroxidase-conjugated streptavidin (LightShift^™^ Chemiluminescent EMSA kit) according to the manufacturer's instructions.

### RNP immunoprecipitation assay

To determine the binding between TTP and *Twist1* or *Snail1* AREs, RNP immunoprecipitation assay was conducted by modification of previously describe [25]. Briefly, cell suspension of NIH:OVCAR3was was incubated in 1% of formaldehyde for 20 min at room temperature. The reaction was stopped with 0.25 M of glycin (pH 7.0), and cells were sonicated in modified RIPA buffer containing protease inhibitors (Roche, Indianapolis, IN, USA). RNP complexes were immunoprecipitated using protein G-agarose beads preincubated with 1 μg of anti-TTP (Sigma) or 1 μg of isotype control (Sigma, St Louis, MO, USA). After crosslinking reversion at 70°C for 45 min, RNA was isolated from the immunoprecipitates and treated with DNAse I (Qiagen, Valencia, CA, USA). cDNA was synthesized from the RNA and *Twist1* 3′UTR and *Snail1* 3′UTR were amplified by PCR using Taq polymerase (Solgent, Daejeon, Korea) and primers (Twist1 UP-CCGCTCGAGCTGGCCTGCAAAAC, Twist1 DOWN-A TAGTTTAGCGGCCGCATGAATGCATTTAGA, Snail1 UP-CCGCTCGAGAGGCAGCTATTTCAG, Snail1 DO WN-ATAGTTTAGCGGCCGCTAATATATAAATTA).

### RNA kinetics, quantitative real-time PCR, and semi-qRT-PCR

For RNA kinetic analysis, we used actinomycin D and assessed TTP mRNA expression using quantitative real-time PCR (qRT-PCR). DNase I-treated total RNA (3 μg) was reverse transcribed using oligo-dT and Superscript II reverse transcriptase (Invitrogen) according to the manufacturer's instructions. qRT-PCR was performed by monitoring in real-time the increase in fluorescence of SYBR Green dye (QIAGEN, Hilden, Germany) using StepOnePlus^™^ Real-time PCR systems (Applied Biosystems). Semi-qRT-PCR was performed using Taq polymerase (Solgent, Daejeon, Korea) and PCR primer pairs (Supplementary Table S1).

### Statistical analysis

For statistical comparisons, *p* values were determined using Student's *t* test or one-way analysis of variance.

## SUPPLEMENTARY MATERIALS TABLES AND FIGURES


