# Supplementary material for: Tristetraprolin suppresses the EMT through the down-regulation of Twist1 and Snail1 in cancer cells
Source: Oncotarget. 2016 Jan 31;7(8):8931–43. doi: 10.18632/oncotarget.7094 (PMC4891015; doi:10.18632/oncotarget.7094)
Supplement: Supplementary file 1 [file oncotarget-07-8931-s001.pdf]

# Tristetraprolin suppresses the EMT through the down-regulation of Twist1 and Snail1 in cancer cells

## Supplementary Materials

## MATERIALS AND METHODS

### Cell morphology and immunofluorescence study

Cells were cultivated on tissue culture dishes and photographed using a Zeiss Axiovert 25 microscope equipped with a Nikon (Tokyo) Coolpix 995 digital camera. For localization of the EMT markers, cells were seeded in coverglass bottomed dish (SPL, 200350) and fixed with paraformaldehyde (4%) followed by protein block serum-free solution (Dako, Glostrup, Denmark) with 0.1% Triton X100. Cells were incubated with anti-human E-cadherin (BD biosciences, 610181), anti-human N-cadherin (BD biosciences, 610920), anti-human vimentin (Cell signaling, 5741), anti-human Twist1 (Abcam, ab49254), and anti-human Snail1 (Abcam, ab167607) antibodies, followed by incubation with the appropriate secondary antibody (anti-mouse or anti-rabbit antibodies conjugated with Alexa-594, Invitrogen or -648, Cell signaling,). The coverslips were mounted using DAPI (4′,6-diamidino-2-phenylindole)-containing anti-fade mounting medium (Vector Lab, Burlingame, CA, USA). Confocal images were acquired in a sequential mode using a 100 × Plan Apochromat NA/1.4 oil objective and the appropriate filter combination on the Olympus 1200 laser-scanning confocal system.

### SDS-PAGE analysis and immunoblotting

Proteins were resolved by SDS-PAGE, transferred onto Hybond-P membranes (Amersham Biosciences Inc.), and probed with appropriate dilutions of rabbit anti-human TTP antibody (Sigma, T5327), anti-E-cadherin (BD biosciences, 610181), anti-N-cadherin (BD biosciences, 610920), anti-vimentin (Cell signaling, 5741), anti-Twist1 (Abcam, ab49254), anti-Snail1 (Abcam, ab167607) and anti-β actin antibodies (Sigma, A2228). Immunoreactivity was detected using the ECL detection system (Amersham Biosciences Inc.). Films were exposed at multiple time points to ensure that the images were not saturated.

### Immunohistochemistry

Immunohistochemical detection of TTP was performed on tissue array slides containing paraffin sections of normal ovarian tissues (OV20810, US Biomax Inc., Rockville, MD, USA) or normal colonic mucosa (BN05011, US Biomax Inc., Rockville, MD, USA). After deparaffinization, anti-human TTP antibody (Santa Cruz, sc-14030) at a 1:100 dilution was applied for 2 h at room temperature. The expression of TTP in the surface epithelium and the mesenchyme of normal ovarian tissues and colonic mucosa was scored semiquantitatively based on the staining intensity. Staining intensity was classified as 0, negative; 1, weak; 2, moderate; 3, strong.

**Supplementary Table S1: PCR primers and oligonucleotides used in this study**

| Name | Sequences (forward and reverse, 5′ to 3′) |
|---|---|
| qTTP | CGCTACAAGACTGAGCTAT,<br>GAGGTAGAACTTGTGACAGA |
| qTwist1 | GCCGGAGACCTAGATGTCATT,<br>CACGCCCTGTTTCTTTGAAT |
| qTwist2 | GCAAGAAGTCGAGCGAAGAT<br>GCTCTGCAGCTCCTCGAA |
| qSnail1 | GCGAGCTGCAGGACTCTAAT,<br>GGACAGAGTCCCAGATGAGC |
| qSnail2 | GCCTCCAAAAAGCCAAACTA,<br>CACAGTGATGGGGCTGTATG |
| qZEB1 | GCCAATAAGCAAACGATTCTG,<br>TTTGGCTGGATCACTTTCAAG |
| qZEB2 | AAGCCAGGGACAGATCAGC<br>CCACACTCTGTGCATTTGAAC |
| qE-cadherin | GAACGCATTGCCACATACAC<br>ATTCGGGCTTGTTGTCATTC |
| qN-cadherin | TCAGTGGCGGAGATCCTACT<br>GTGCTGAATTCCCTTGGCTA |
| qVimentin | TGTCCAAATCGATGTGGATGTTTC,<br>TTGTACCATTCTTCTGCCTCCTG |
| qCDC34 | TGACCAAGATGTGGCACCCTAACA,<br>TGATGTCTGTGTACTCCCGATCCT |
| qGAPDH | ACATCAAGAAGGTGGTGAAG,<br>CTGTTGCTGTAGCCAAATTC |
| c-Fos | ACGCAGACTACGAGGCGTCA,<br>TTCACAACGCCAGCCCTGGA |
| VEGF | CGAAGTGGTGAAGTTCATGGATGT,<br>TCACCGCCTCGGCTTGTC |
| Twist1–3′UTR-U | CCGCTGGAGCAGGCGGAGCCCCCCACCCC |
| Twist1–3′UTR-D | ATAAGAATGCGGCCGCTTTTTCTCTAAATTTTTTAT |
| Snail1–3′UTR-U | CCGCCCTCGAGGCTCCCTCTTCCT |
| Snail1–3′UTR-D | ATAAGAATGCGGCCGCAATATATAAATTAACTGCTT |
| Oligo-Twist1 ARE1 | TCGAGACTTAAAATACAAAAAACAACATTCTATTTATTTATTGAGGACCCATGGTAA<br>AATGCAAAGC,<br>GGCCGCTTTGCATTTTACCATGGGTCCTCAATAAATAAATAGAATGTTGTTTTTTGT<br>ATTTTAAGTC |
| Oligo-Twist1<br>ARE1 MUT | TCGAGACTTAAAATACAAAAAACAACATTCTAGCAGCATTGAGGACCCATGGTAA<br>AATGCAAAGC,<br>GGCCGCTTTGCATTTTACCATGGGTCCTCAATGCTGCTAGAATGTTGTTTTTTGTAT<br>TTTAAGC |
| Oligo-Twist1 ARE2 | TCGAGTTTGTAAATATCTTTGTATATTTTTCTGCAATAAATAAATATAAAAAATTTAG<br>AGAAAAAGC,<br>GGCCGCTTTTTCTCTAAATTTTTTATATTTATTTATTGCAGAAAAATATACAAAGATA<br>TTTACAAAC |

| | |
|---|---|
| Oligo-Snail1 ARE1 | TCGAGTGGGGTGGCACCTGTTTCCCGGGCAATTTAACAATGTCTGAAAAGGGACT<br>GTGAGGC,<br>GGCCGCCTCACAGTCCCTTTTCAGACATTGTTAAATTGCCCGGGAAACAGGTGCC<br>ACCCCAC |
| Oligo-Snail1 ARE2 | TCGAGTTTACATTTTTAAAGGTACACTGGTATTTATATTTCAAACATTTTGTATCAAG<br>GAGC,<br>GGCCGCTCCTTGATACAAAATGTTTGAAATATAAATACCAGTGTACCTTTAAAAATG<br>TAAAC |
| Oligo-Snail1 ARE3 | TCGAGGTTATATGTACAGTTTATTGATATTCAATAAAGCAGTTAATTTATATATTAAAA<br>AGC,<br>GGCCGCTTTTTAATATATAAATTAACTGCTTTATTGAATATCAATAAACTGTACATATA<br>ACC |
| Oligo-Snail1 ARE3 MUT | TCGAGGTTATATGTACAGTTTATTGATATTCAATAAAGCAGTTAAGCATATATTA<br>AAAAGC,<br>GGCCGCTTTTTAATATAGCTTAACTGCTTTATTGAATATCAATAAACTGTACATATAA<br>C C |
| Oligo-Twist1 ARE1 | TCGAGACTTAAAATACAAAAAACAACATTCTATTTATTTATTGAGGACCCATGGTAA<br>AATGCAAAGC,<br>GGCCGCTTTGCATTTTACCATGGGTCCTCAATAAATAAATAGAATGTTGTTTTTTGT<br>ATTTTAAGTC |

The underlined sequences are restriction enzyme sites.

**Supplementary Table S2: TTP expression in TTP in the surface epithelium and the mesenchyme of normal ovarian tissues and colonic mucosa**

| | *n* | Staining intensity | | *p* value |
|---|---|---|---|---|
| | | Epithelium | Mesenchyme | |
| Normal ovarian tissue | 16 | 2.56 ± 0.04 | 0.25 ± 0.03 | < 0.0001 |
| Normal colonic mucosa | 48 | 2.20 ± 0.01 | 0.27 ± 0.01 | < 0.0001 |

Mean ± S.E.M.

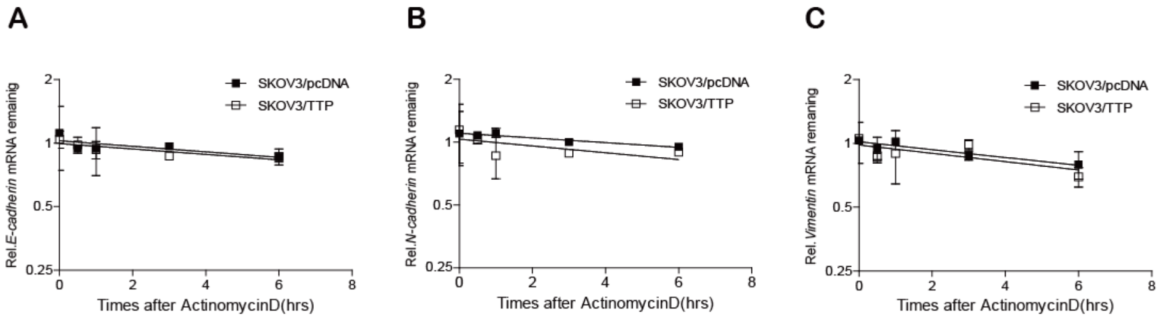


**Supplementary Figure S1: Overexpression of TTP does not affect the mRNA stabilities of the EMT markers *E-cadherin*, *N-cadherin*, and *vimentin*.** (**A–C**) TTP destabilizes the mRNAs of *Twist1* and *Snail1*. SKOV3 cells were transfected with pcDNA6/V5-TTP or pcDNA6/V5 for 24 h. The expression of *E-cadherin* (A), *N-cadherin* (B), and *vimentin* (C) mRNAs in SKOV3 cells was determined by qRT-PCR at the indicated times after the addition of 5 μg/ml actinomycin D. Data are presented as the mean ± SD ($n = 3$). ns, not significant.

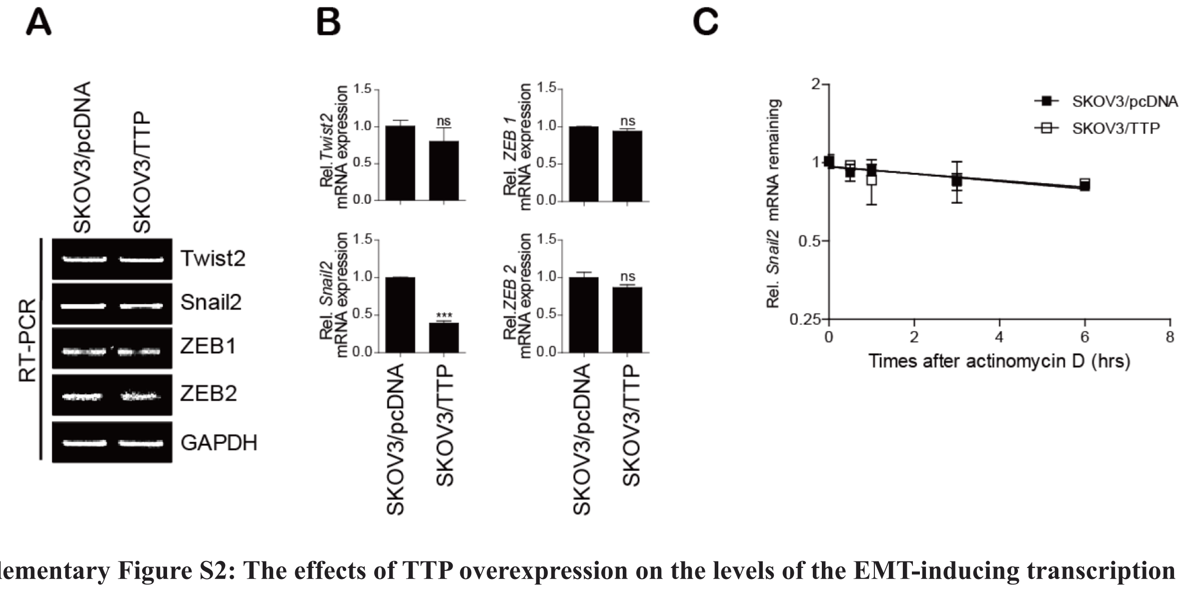


**Supplementary Figure S2: The effects of TTP overexpression on the levels of the EMT-inducing transcription factors *Twist2*, *Snail2*, *ZEB1*, and *ZEB2*.** SKOV3 cells were transfected with pcDNA6/V5-TTP or pcDNA6/V5 for 24 h. (**A–B**) The levels of *Twist2*, *Snail2*, *ZEB1*, and *ZEB2* were determined by semi-qRT-PCR (A) and qRT-PCR (B). Data are presented as the mean ± SD ($n = 3$) (***$p < 0.001$). ns, not significant. (**C**) TTP does not enhance the decay of *Snail2* mRNA. Expression of *Snail2* mRNA in SKOV3 cells was determined by qRT-PCR at the indicated times after the addition of 5 μg/ml actinomycin D. Data are presented as the mean ± SD ($n = 3$). ns, not significant.

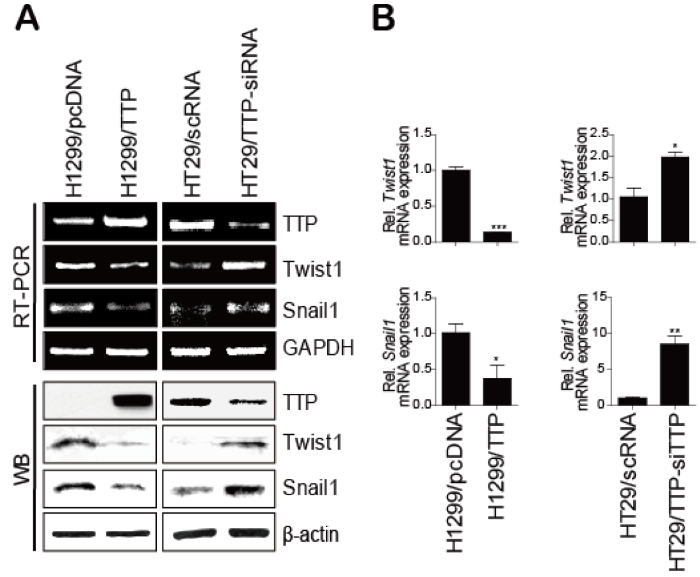


**Supplementary Figure S3: TTP inhibits the expression of *Twist1* and *Snail1* in H1299 and HT29 cells.** H1299 cells were transfected with pcDNA6/V5-TTP (H1299/TTP) or pcDNA6/V5 (H1299/pcDNA) for 24 h. In addition, HT29 cells were transfected with TTP-specific (HT29/TTP-siRNA) or scRNA (HT29/scRNA) for 24 h. (**A**) The levels of *TTP*, *Twist1*, and *Snail1* were determined by semi-qRT-PCR (A, top), Western blot (A, bottom), and qRT-PCR. (**B**) Data are representative of three experiments. Data are presented as the mean ± SD ($n = 3$) (*$p < 0.05$; **$p < 0.01$; ***$p < 0.001$).

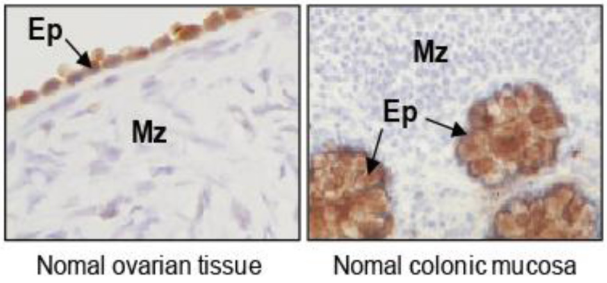


**Supplementary Figure S4: TTP expression levels are high in the epithelium but low in the mesenchyme.** Representative immunohistochemical staining of TTP in normal ovarian tissue and colonic mucosa. Ep, epithelium; Mz, mesenchyme.

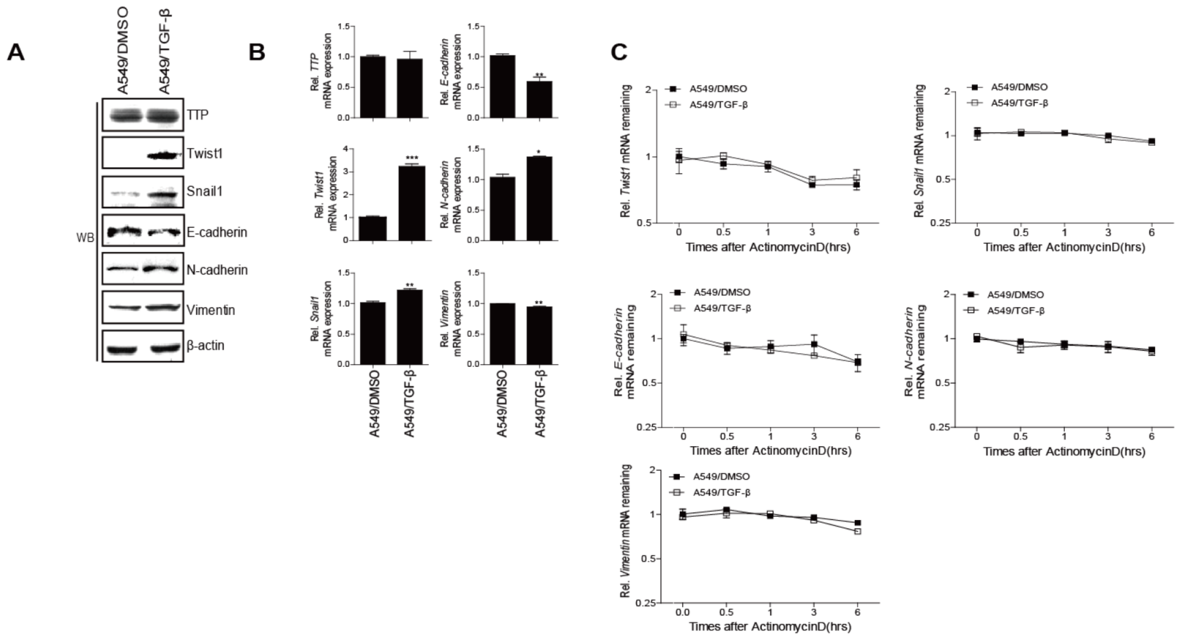


**Supplementary Figure S5: TGF-β induces the expression of EMT markers without affecting the TTP level and mRNA stability in A549 cells.** A549 cells were stimulated with 10 ng/ml TGF-β for 24 h. (**A–B)** The levels of *TTP, Twist1, Snail1, E-cadherin, N-cadherin* and *vimentin* mRNAs were determined by (A) Western blot and (B) qRT-PCR. Data are presented as the mean ± SD ($n$ = 3) (*$p < 0.05$; **$p < 0.01$; ***$p < 0.001$). (**C**) The expression of *Twist1*, *E-cadherin*, *N-cadherin*, and *vimentin* mRNAs in A549 cells was determined by qRT-PCR at the indicated times after the addition of 5 µg/ml actinomycin (**D**) Data are presented as the mean ± SD ($n$ = 3).

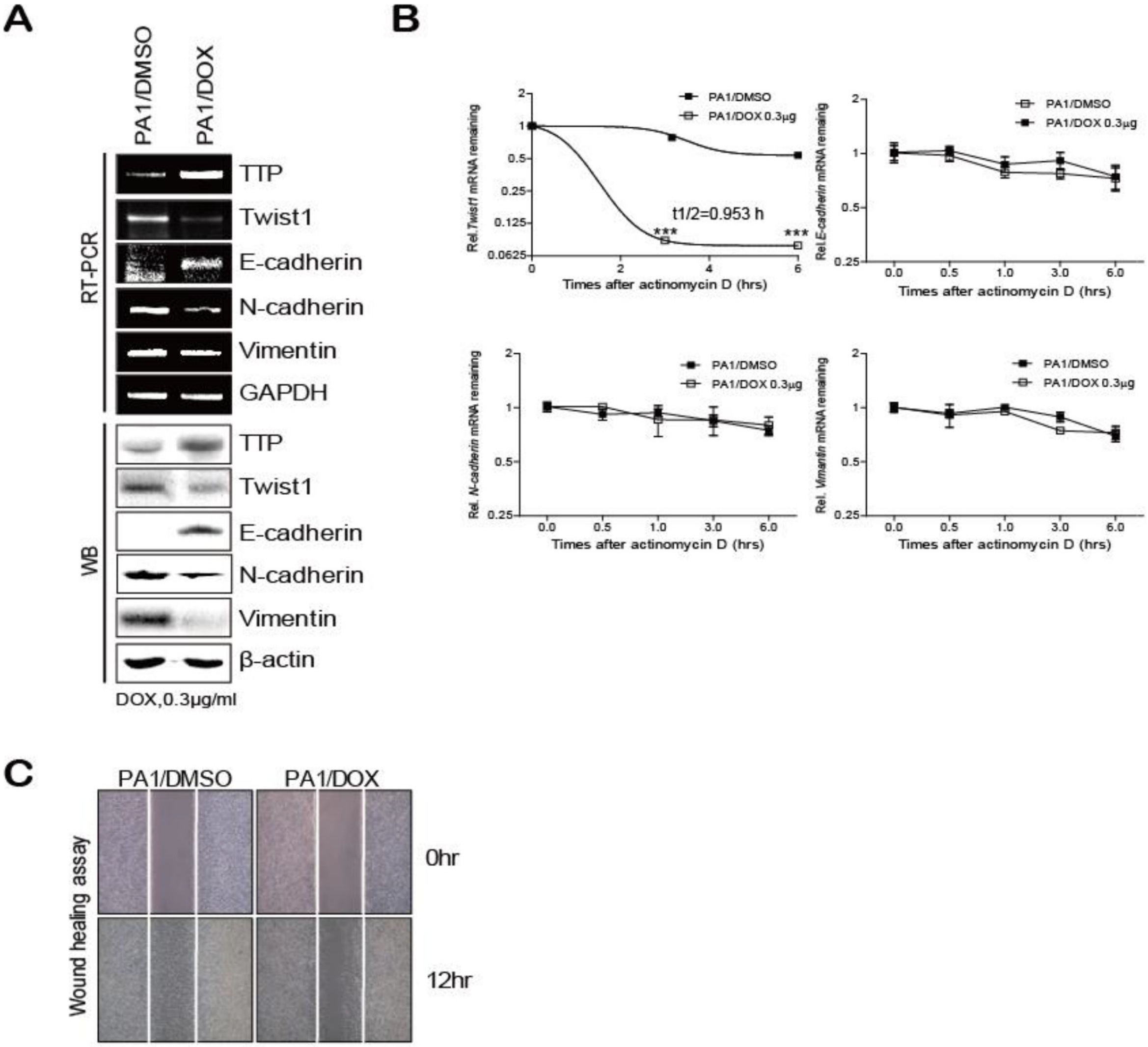


**Supplementary Figure S6: DOX induces TTP expression and an epithelial phenotype.** PA1 ovarian cancer cells were treated with 0.3 μg/ml of DOX for 24 h. (**A**) The levels of *TTP*, *Twist1*, *E-cadherin*, *N-cadherin*, and *vimentin* were determined by semi-qRT-PCR (top) and Western blot (bottom). (**B**) The expression of *Twist1*, *E-cadherin*, *N-cadherin*, and *vimentin* mRNAs in PA1 cells was determined by qRT-PCR at the indicated times after the addition of 5 μg/ml actinomycin (**D**) Data are presented as the mean ± SD ($n = 3$). (**C**) Wound-healing assay. The wounded areas of PA1 cells were examined under × 20 magnification.
